# Towards a European platform for Rare Diseases Registries

**DOI:** 10.1186/1750-1172-9-S1-O6

**Published:** 2014-11-11

**Authors:** Ciarán Nicholl

**Affiliations:** 1 JRC, Ispra, EU Commission, EU

## 

The Joint Research Centre (JRC) is a Directorate-General (DG) of the European Commission (EC), it was established in 1957 and its mission is to provide independent, evidence-based scientific and technical support throughout the EU policy cycle. Health is a key European policy area and research on Rare Diseases (RD) is identified as a priority in both the Commission Communication on RD: Europe's challenges COMM (2008) 679 final and the Council Recommendation on an action in the field of RD (2009/C 151/02).

The specificities of RD – limited number of patients and scarcity of information – single them out as a unique domain for which an action at European level has high potential added value. For this reason and in the context of developing a long-term EU strategy in the area of health information including RD data collection, DG Health and Consumers (SANCO) and the JRC started an initiative aimed to improve the sustainability of the results achieved over the past years. Taking into account that the current fragmentation of data sources is a key obstacle to steering EU policy and to performing high quality research (e.g. conducting epidemiological, clinical, pharmacological studies) and in the end to advancing knowledge on RD, the two DGs agreed on the development of the European Platform for RD registries. The main purpose of the Platform which will be established at the JRC is to support the existing registries by promoting their interoperability and accessibility, by improving RD data comparability and reliability. It will also support the development of new registries. This 'hub' will facilitate data analysis within and across many RD and across Europe and provide sound information on RD for policy, clinical trials and research. The Platform is intended to provide a central access point for information on RD patients registries for all stakeholders – health professionals, researchers, patients, public health authorities, industry, etc. Thus the benefits of collaboration and maximisation of limited resources are most obvious in a concerted action with the final aim of improving the quality of care and the quality of life for RD patients.

